# Dynamics of photonic toroidal vortices mediated by orbital angular momenta

**DOI:** 10.1126/sciadv.adz0843

**Published:** 2025-09-26

**Authors:** Xin Liu, Nianjia Zhang, Qian Cao, Jinsong Liu, Chunhao Liang, Qiwen Zhan, Yangjian Cai

**Affiliations:** ^1^ Shandong Provincial Engineering and Technical Center of Light Manipulations and Shandong Provincial Key Laboratory of Optics and Photonic Device, School of Physics and Electronics, Shandong Normal University, Jinan 250014, China.; ^2^ Collaborative Innovation Center of Light Manipulations and Applications, Shandong Normal University, Jinan 250358, China.; ^3^ School of Optical-Electrical and Computer Engineering, University of Shanghai for Science and Technology, Shanghai 200093, China.; ^4^ Zhejiang Key Laboratory of 3D Micro/Nano Fabrication and Characterization, Department of Electronic and Information Engineering, School of Engineering, Westlake University, Hangzhou, Zhejiang 310030, China.

## Abstract

The dynamics of fluidic vortex rings have long captivated researchers because of their complex behavior despite simple structure. Photonic toroidal vortices constitute a family of three-dimensional, spacetime nonseparable structured light fields carrying transverse orbital angular momentum (OAM). However, as solutions to the dispersive Maxwells’ equations, these wave packets do not survive nondispersive propagation, and their dynamics remain elusive. In this article, the dynamics of photonic toroidal vortices under various dispersion regimes, mediated by transverse and longitudinal OAM, are investigated through simulations and experiments. The results reveal that the motion of a toroidal vortex is strongly affected by longitudinal OAM. The swirling flow destabilizes the toroidal structure under dispersion and induces topological transformations of vortex lines, characterized by annihilation and subsequent reformation in vacuum. The renascent toroidal vortex exhibits robust propagation in vacuum while maintaining its structure. These findings are supported by experimental validation and highlight the potential of photonic toroidal vortices as controllable channels for directional energy and information transfer.

## Teaser

Toroidal light vortices twist, break, and reform in spacetime, revealing routes for controlling and transporting light.

## INTRODUCTION

Toroidal vortices, also known as vortex rings, are fascinating three-dimensional (3D) torus-shaped structures characterized by vortical flow confined around a closed vortex line. Such flow patterns are frequently encountered in natural contexts and fluid mechanics (*1*, *2*), manifest in various forms, ranging from the turbulent flows of liquids and gases (*3*–*5*) such as smoke rings and cavitation bubbles, to thermally induced formations in mushroom clouds (*4*), and biologically produced vortices associated with microscale locomotion (*5*), seed dispersal (*6*, *7*), and cardiovascular flow (*8*). Despite the apparent simplicity of toroidal vortices structure, their evolution exhibits intriguing complexity and has long motivated extensive research aimed at elucidating these dynamics under various flows (*9*–*13*).

**Fig. 1.**
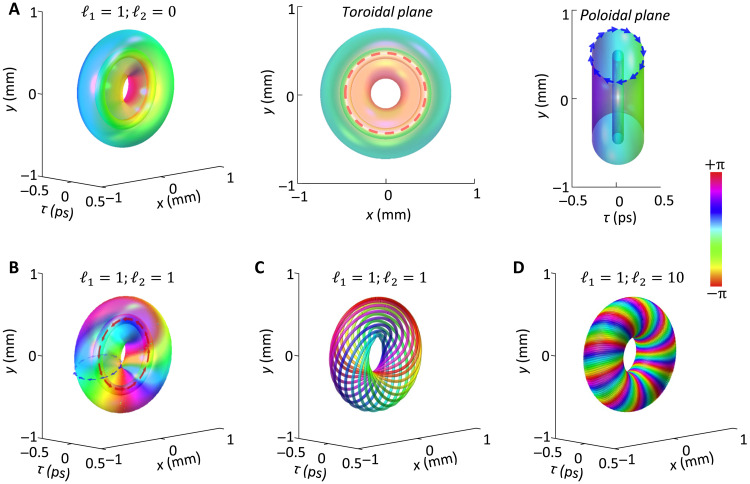
3D profile and phase map of photonic toroidal vortices. (**A**) Iso-intensity profile and phase map of a toroidal vortex of ${𝓁}_{1}=1$ and ${𝓁}_{2}=0$ in different perspective views. (**B**) Iso-intensity profile and phase map of a photonic toroidal vortex of ${𝓁}_{1}=1$ and ${𝓁}_{2}=1$. (**C**) and (**D**) The equiphase representation of a hopfion wave packet with different ${𝓁}_{2}$. The color denotes phase. The parameters are $\lambda_{0}=800\;\text{nm}$ $w_{\tau }=$1 ps, $w_{x}=1$ mm, $w_{0}=$ 0.25. $r_{0}=0.5$ mm.

In the realm of electromagnetics, two types of toroidal light vortices have attracted wide attention since their experimental creations. One is the vector toroidal pulse, which is visualized as a torus made of electric/magnetic field lines (*14*–*17*), exhibits nontrivial topological textures (*18*, *19*) and propagation-robust dynamics (*20*–*22*). Another representative structure is the scalar photonic toroidal vortex (*23*), characterized by a closed-loop spatiotemporal optical vortex (STOV) (*24*–*28*). This configuration gives rise to a 2π phase variation around the surface of a torus in the poloidal plane, thereby yielding a transverse orbital angular momentum (OAM) density that is tangent to the vortex line, as shown in Fig. 1. A toroidal vortex wave packet is an approximate solution to the time-dependent wave equation under anomalous dispersion, but it exhibits strongly unstable evolution in free space (*23*, *29*). A toroidal vortex carrying a toroidal swirl forms a scalar optical hopfion topology (*30*), which encompasses both transverse and longitudinal OAM. It has been proven that the coupling between these OAMs within a toroidal vortex leads to entirely distinct topological properties in high-harmonic generation (*31*). It is amazing that fluid vortex rings are capable of holding their morphology for a long time in a calm medium. However, although the photonic toroidal vortices can now be readily synthesized in free space, their robust free propagation is still out of reach, and their dispersive spreading and motion under the interactions of transverse and longitudinal OAMs are not yet investigated.

Understanding the dynamics and mutual interaction of various types of vortices is a key ingredient in clarifying and taming high-dimensional light. In this study, we reveal that spatiotemporal toroidal vortices exhibit nontrivial dispersive dynamics, mediated by the coupling between their transverse and longitudinal OAM. In the absence of longitudinal OAM, the toroidal vortex evolves similarly to a conventional STOV pulse. However, the inclusion of a longitudinal OAM disturbs the flow of toroidal vortex, fundamentally alters the dispersive behavior, leading to several distinctive phenomena: (i) The toroidal vortex becomes highly unstable in both normally and anomalously dispersive media; (ii) in vacuum, the vortex line undergoes breakdown and subsequent reconstruction during propagation, and the renascent toroidal vortex robustly preserves its morphology over extended distances. These findings are confirmed in the experiments.

## RESULTS

### Numerical simulation results and analysis

The complex field of a toroidal vortex wave packet at z=0 can be expressed as

$$\begin{array}{c} \Psi \left(x,y,\tau \right)={\left(\frac{\sqrt{2}\rho_{⊥}}{w_{0}}\right)}^{|\ell_{1}|}\text{exp}\left(-\frac{\rho_{⊥}^{2}}{w_{0}^{2}}\right)L_{p}^{|\ell_{1}|}\left(\frac{2\rho_{⊥}^{2}}{w_{0}^{2}}\right)\text{exp}\left[-i\left(\ell_{1}\varphi_{⊥}+\ell_{2}\gamma_{s}\right)\right] \end{array}$$ (1)

where $L_{p}^{|\ell_{1}|}\left(\cdot \right)$ is the associated Laguerre polynomial. $\rho_{⊥}=\sqrt{{\left(r_{s}-r_{0}\right)}^{2}+\tau^{2}}$, $\varphi_{⊥}={\text{tan}}^{-1}\left[\tau /\left(r_{s}-r_{0}\right)\right]$ are defined in the poloidal plane and $r_{s}=\sqrt{x^{2}+y^{2}}$, $\gamma_{s}={\text{tan}}^{-1}\left(y/x\right)$ are defined in the toroidal plane; $r_{0}$ is a constant that quantifies the radius of the toroidal vortex line; ${𝓁}_{1}$ and ${𝓁}_{2}$ are the winding numbers that quantify the twist number of helicoid phases at the poloidal and toroidal plane, respectively. Figure 1A displays the 3D structure of a photonic toroidal vortex with ${𝓁}_{2}=0$, which has a closed vortex line. Since ${𝓁}_{2}=0$, the poloidal energy flow pattern is identical to that of a standard STOV (see sections S1 and S2). The averaged transverse OAM ($\left\langle {\hat{L}}_{x}\right\rangle$ and $\left\langle {\hat{L}}_{y}\right\rangle$, where ⟨·⟩ denotes the averaged as defined in section S3) carried by a toroidal vortex depends on the dispersion regimes, which in turn gives rise to distinct energy flow patterns (see sections S1 and S2). When ${𝓁}_{2}\neq 0$, Eq. 1 describes the wave function of a scalar optical hopfion, whose phase structure can be visualized as a torus formed by nested equiphase lines, as shown in Fig. 1 (B to D) for different ${𝓁}_{2}$. A toroidal vortex with ${𝓁}_{2}\neq 0$ simultaneously exhibits distinct energy flow, governed by ${𝓁}_{1}$ and $\beta_{2}$ in the poloidal plane, and a swirl energy flow induced by the longitudinal OAM $\left\langle {\hat{L}}_{\tau }\right\rangle \propto {𝓁}_{2}\neq 0$ in the toroidal plane (section S3). The poloidal flow (dependent on dispersions) interacts with toroidal swirl, yielding a complex energy flow (section S2). To investigate the interplay between transverse and longitudinal OAM in the dynamics of a toroidal vortex, it is important to consider a balanced regime of dispersion and diffraction, characterized by $|\beta_{2}|=k_{0}^{-1}$, to avoid excessive effects from either. An approximate relationship $r_{0}\sim w_{0}\sqrt{|\;\ell_{2}\;|/2}$ then provides a basis for ruling the propagation dynamics of photonic toroidal vortices governed jointly by dispersion and diffraction through the coupling of transverse and longitudinal OAM (section S4).

**Fig. 2.**
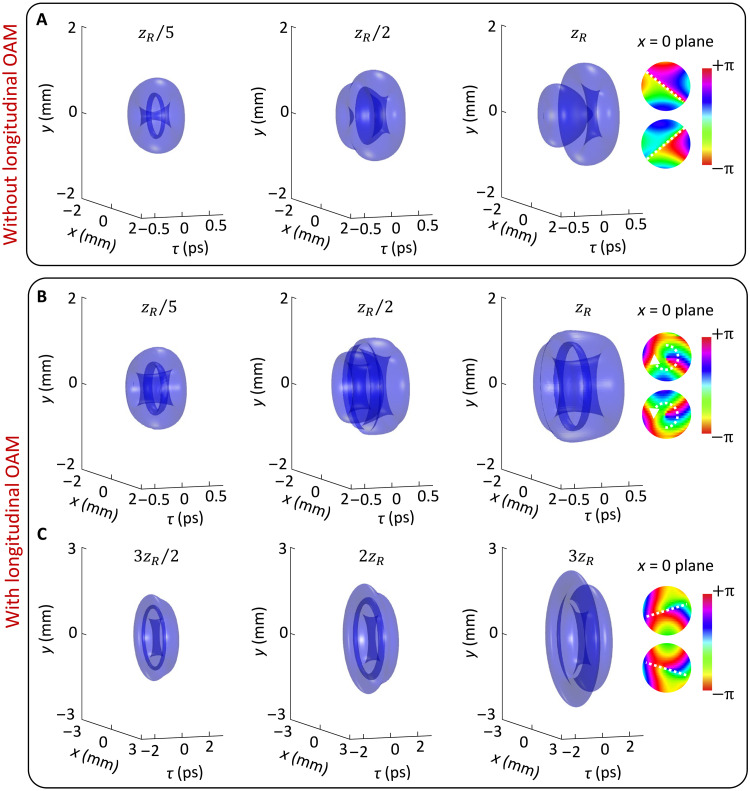
Iso-intensity surfaces (10% of the peak intensity) of toroidal vortex wave packet with $\ell_{1}=1$ propagating in a normally dispersive medium with $\beta_{2}=k_{0}^{-1}$. (**A**) ${𝓁}_{2}=0$ and (**B**) and (**C**) ${𝓁}_{2}=10$. Other parameters are the same as in Fig. 1.

**Fig. 3.**
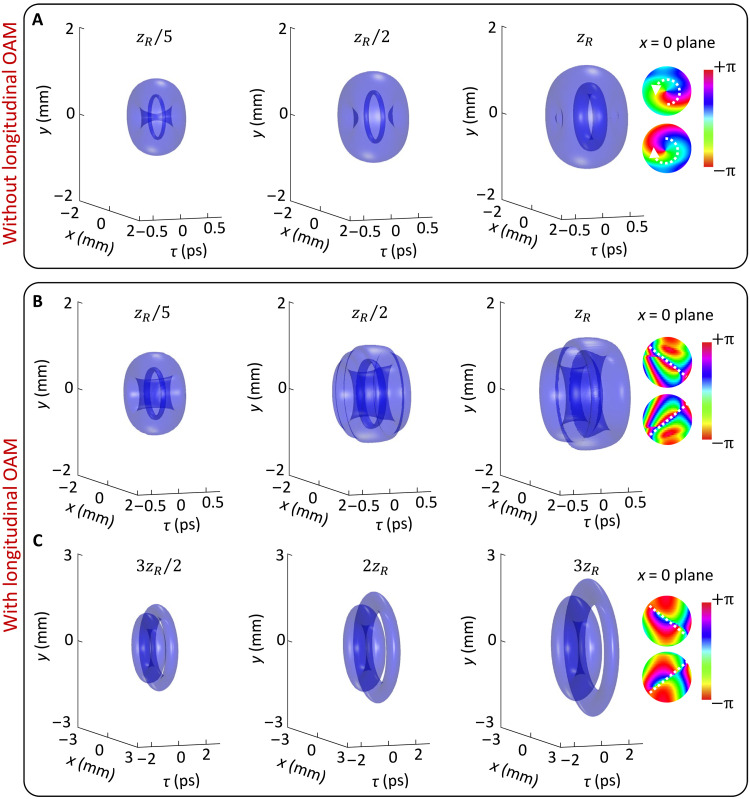
Iso-intensity surfaces (10% of the peak intensity) of toroidal vortex wave packet with $\ell_{1}=1$ propagating in an anomalously dispersive medium with $\beta_{2}=-k_{0}^{-1}$. (**A**) ${𝓁}_{2}=0$ and (**B**) and (**C**) ${𝓁}_{2}=10$. Other parameters are the same as in Fig. 1.

**Fig. 4.**
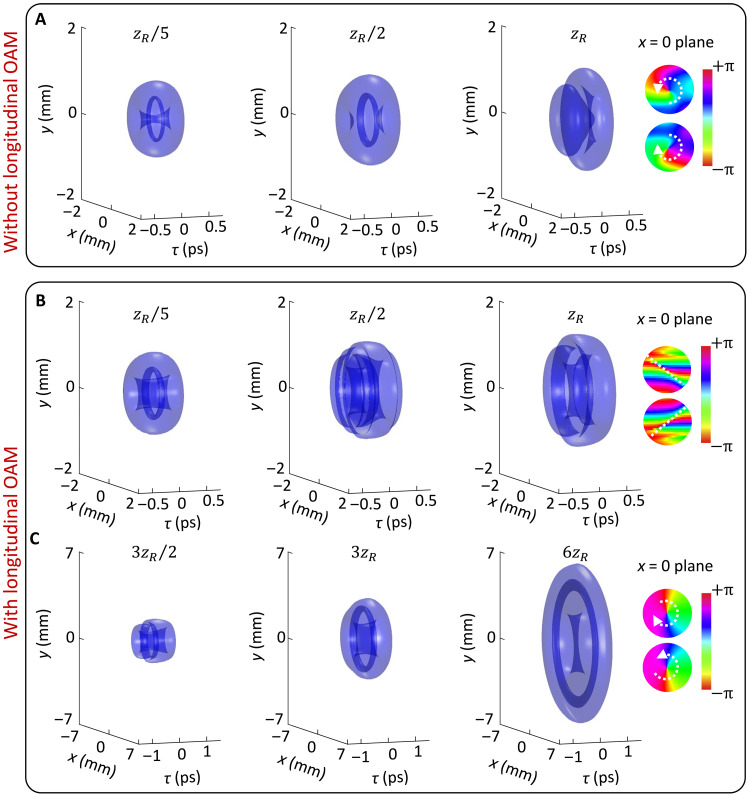
Iso-intensity surfaces (10% of the peak intensity) of toroidal vortex wave packet with $\ell_{1}=1$ propagating in vacuum with $\beta_{2}=0$. (**A**) ${𝓁}_{2}=0$ and (**B**) and (**C**) ${𝓁}_{2}=10$. Other parameters are the same as in Fig. 1.

In simulations, we use the angular spectrum relation (see section S3) to numerically calculate the full spacetime wave packet of toroidal vortices in dispersive media. Figures 2 to 4A show the propagation dynamics of toroidal vortices without a longitudinal OAM under different dispersion cases. In all cases, the ring-shaped structure of the toroidal vortex collapses during propagation as a result of spatial diffraction within the toroidal plane. In contrast, for toroidal vortices carrying longitudinal OAM, the ring-shaped structure is preserved, as shown in Figs. 2 to 4 (B and C). This difference arises from the fact that the longitudinal OAM induces a swirling flow in the toroidal plane, which helps preserve the ring-shaped structure. However, this swirling flow interacts with the poloidal flow, giving rise to more complex spatiotemporal dynamics, as will be discussed in detail below.

In a normally dispersive medium ($\beta_{2}=k_{0}^{-1}$), the vortex line carried by the toroidal vortex without longitudinal OAM in Fig. 2A becomes stretched, finally causing it to split and peel off two separated pieces in time. The phase difference between the two pieces approaches π near the fissure because of the “saddle” energy flow (see sections S1 and S2). However, in Fig. 2B, the toroidal vortex with longitudinal OAM initially breaks into two separate pieces over a short propagation distance. After that, the vortex line quickly reconstructs, and the entire wave packet exhibits a well-defined toroidal structure. The interaction between saddle- and spiral-shaped energy flows leads to a polarity reversal of the reconstructed vortex line. While the structure at this position is highly unstable, as propagation further proceeds in Fig. 2C, the toroidal vortex undergoes a second breakup, splitting into two independent pieces. In contrast to the evolution in Fig. 2A, this peeling off occurs in a reversed direction because of the polarity reversal of the vortex line.

Figure 3 shows the dynamics of toroidal vortices propagating in an anomalously dispersive medium ($\beta_{2}=-k_{0}^{-1}$). For the toroidal vortex without longitudinal OAM (Fig. 3A), its poloidal energy flow shows a spiral pattern, and thus, the entire morphology is preserved during propagation. However, the introduction of longitudinal OAM disturbs this stable regime. As shown in Fig. 3 (B and C), the vortex line stretches and eventually vanishes, leading to a complete splitting of the toroidal vortex into two separate components. Notably, the direction of the breakup is reversed compared to that observed in the normally dispersive case (Fig. 2).

We now examine the role of longitudinal OAM on the dynamics of toroidal vortices in vacuum ($\beta_{2}=0$). Figure 4A shows the toroidal vortex without longitudinal OAM in the process of collapsing, similar to the cases of Fig. 2A. As the poloidal energy flow is confined to the radial direction, propagation of the toroidal vortex without longitudinal OAM in vacuum over a Rayleigh length will introduce notable distortions. In comparison with Fig. 4A, the results in Fig. 4B indicate that the presence of longitudinal OAM further accelerates the structural distortion. Even more interestingly in Fig. 4C, as propagation proceeds beyond a Rayleigh length, the vortex line gradually reconstructs, and the entire wave packet reforms into a well-defined toroidal structure. The reconstructed toroidal vortex contains a polarity-reversed vortex line and maintains its morphology over extended propagation in vacuum (see movie S1).

### Experimental observation of toroidal vortices dynamics mediated by transverse and longitudinal OAM

**Fig. 5.**
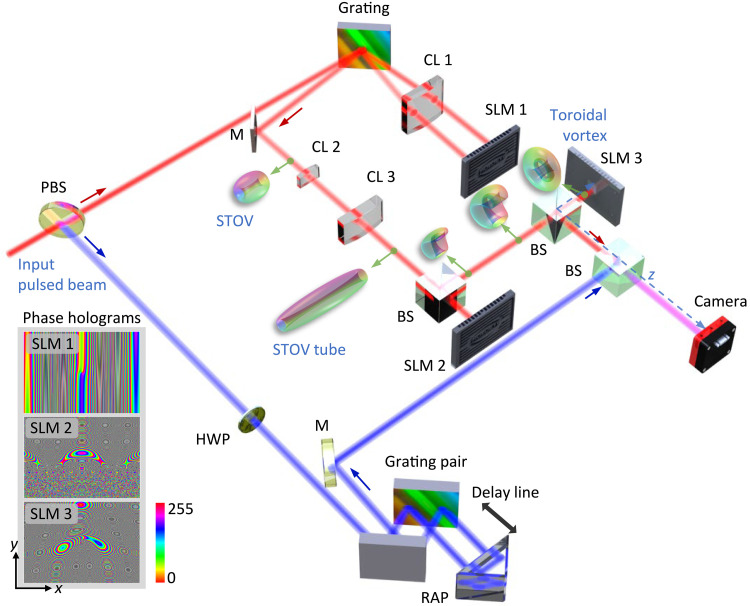
Schematic of the experimental setup to synthesize and propagate photonic toroidal vortices. PBS, polarization beam splitter; HWP, half-wave plate; CL 1–3, cylindrical lens; SLM 1–3, spatial light modulator; BS, beam splitter; M, mirror; RAP, right-angle prism. The phase pattern imparted by SLM 1 and SLM 3 includes an additional group delay dispersion phase and a helical phase, respectively.

The experimental realization of a photonic toroidal vortex can proceed by bending an STOV tube in a conformal mapping system (*23*, *32*). We conducted an experiment that combines 2D pulse shaping with conformal mapping as illustrated in Fig. 5, to verify the simulation results. First, an input pulse centered at 1012 nm with an ~20-nm bandwidth (section S5) is incident onto a 2D pulse shaper. The pulse shaper consists of a grating (1200 lines/mm), a cylindrical lens with a 10-cm focal length along the 𝑥 axis, and a helical phase pattern [Fig. 5 left, in which a group delay dispersion (GDD) phase is added]. The modulated pulsed beam is then reflected back, and the grating reconstructs the pulse, generating a near-field STOV pulse with a spatial width of ~250 μm. The STOV pulse then travels through an afocal cylindrical beam expander (the ratio of focal length is 2:14 cm) and stretches in the direction of the vortex line, forming an STOV tube along the *x* axis. Second, we perform Cartesian to log-polar coordinate transformation to this STOV tube, using dual-phase holograms according to conformal mapping theory. In the second stage, the second spatial light modulator (SLM) 2 transforms the STOV tube into a ring-shaped toroidal vortex after propagating d = 23 cm. This is followed by another phase hologram that further shapes the field and applies an additional helical phase of ${𝓁}_{2}=10$, collimating it and completing the afocal transformation. This process ultimately forms a swirl photonic toroidal vortex immediately after the third SLM 3 (at *z* = 0). The propagated toroidal vortex at *z* = 0.8 m is characterized by off-axis interference with a dechirped reference pulse (~100 fs). We note that the temporal dispersion of a wave packet can be equivalently investigated by managing pulse chirp, as temporal diffraction affects the toroidal vortex wave packet similarly to how group velocity dispersion (GVD) influences optical pulses (*33*, *34*). Accordingly, we propagate the generated toroidal vortex in “virtual” dispersive media, where the effective dispersion is readily reprogrammable by modifying the GDD phase using SLM 1; more experimental details are given in Materials and Methods. Last, the 3D complex field of the wave packet can be reconstructed from a series of time-delayed interference fringes (section S5).

**Fig. 6.**
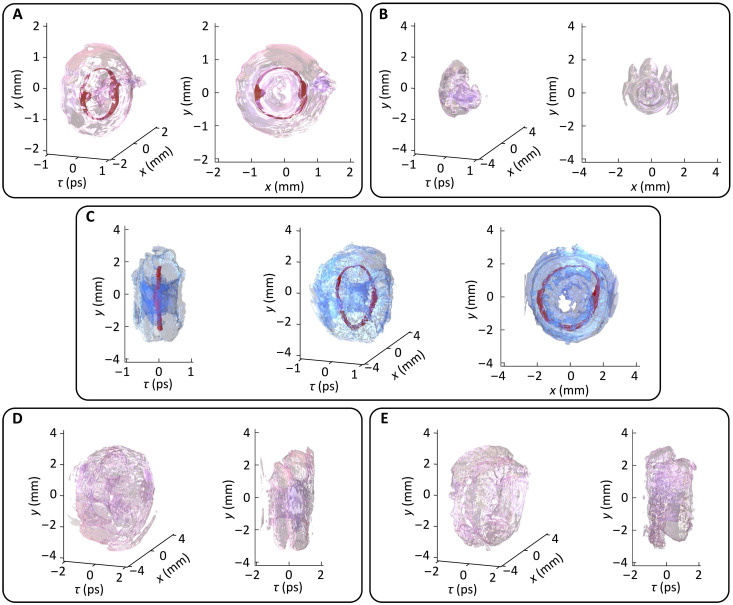
Experimental observations of dispersive dynamics of photonic toroidal vortices. (**A**) 3D iso-intensity profile in different views of the synthesized toroidal vortex source with ${𝓁}_{1}=1$. (**B**) Iso-intensity profile in different views of a toroidal vortex spreading in vacuum without longitudinal OAM. (**C**) 3D iso-intensity profile in different views of a renascent toroidal vortex with ${𝓁}_{2}=10$ spreading in vacuum. (**D**) Iso-intensity profile in different views of a toroidal vortex with ${𝓁}_{2}=10$ spreading in a normal dispersion with a GDD = +0.15 ps^2^. (**E**) 3D iso-intensity profile in different views of a toroidal vortex with ${𝓁}_{2}=10$ spreading in an anomalous dispersion with a GDD = −0.15 ps^2^. The iso-values are set to (A) 2.5% and (B) to (E) 1% of the peak intensity. The results in (B) to (E) are measured at 0.8 m, approximately 3$z_{R}$.

Figure 6A shows the experimental results of the synthesized toroidal vortex wave packet of ${𝓁}_{1}=1$ at the source (*z* = 0 reflected from the SLM 3). For better visualization, semitransparency is applied to the outer torus so that the inside ring-shaped vortex line is clearly shown. The vortex line is colored in red to make the isolated vortex line stand out. The spatial diameter in the toroidal plane of the toroidal vortex is 1 mm and 250 μm in the poloidal plane, corresponding to the Rayleigh length $z_{R}$ = 245 mm. Direct propagation of such a toroidal vortex wave packet in air over long distances results in severe distortions. As shown in Fig. 6B, the wave packet undergoes splitting and collapse, leading to the loss of its toroidal structure. For a toroidal vortex carrying longitudinal OAM of ${𝓁}_{2}=10$, as seen in Fig. 6C, a well-defined toroidal structure is reconstructed and the isolated vortex line reappears. The effect of material dispersion on the wave packets is virtually implemented by adjusting the GDD phase on the pulse shaper. Figure 6 (D and E) shows the toroidal vortices with ${𝓁}_{2}=10$ under different dispersion cases. The change in GDD value corresponds to a propagation distance of 0.8 m in a dispersive medium of $|\beta_{2}|=$160 fs^2^/mm (approximately $|\beta_{2}|=k_{0}^{-1}$). In these cases, the wave packets split reversely in time, and the vortex lines disappear. The renascent toroidal vortex in free space is distinguished by torus colors different from those of the dispersion-associated breakdown structures. The detailed evolution of toroidal vortices with increasing topological charge ${𝓁}_{2}$ values and propagation distances are also provided and discussed in section S6 and movie S2. All experimental results are consistent with the simulations presented in Figs. 2 to 4.

## DISCUSSION

In summary, we demonstrate the propagation dynamics of a class of spatiotemporal toroidal vortex mediated by both transverse and longitudinal OAM. Without the support of longitudinal OAM, the toroidal vortex follows typical STOV propagation dynamics with its structure rapidly deforms and collapses in free space. Toroidal vortices endowed with longitudinal OAM exhibit propagation instability in both normally and anomalously dispersive media, wherein the disappearance of the vortex line results in temporal splitting and disintegration of the wave packet. What is more interesting is that a photonic toroidal vortex with swirling flow in vacuum undergoes the vanishing and subsequent reconstruction of its vortex line. The renascent toroidal vortex can then stably propagate over an arbitrarily long distance in free space (section S7). Owing to its propagation-robust morphology, the photonic toroidal vortex can serve as an effective energy carrier for realizing directional information transfer. These findings hold potential implications for novel photonic topologies engineering as well as for the development of robust optical encoding, free-space information transmission, remote sensing, and ultrafast light-matter interactions.

Last and notably, some of the phenomena described for the propagation dynamics of toroidal vortices of light have been observed also in fluidics. A toroidal vortex evolved in an anomalously dispersive medium exhibits a spiral energy flow in the poloidal plane—resembling that of a vortex ring in an inviscid incompressible fluid—demonstrating a structurally sustained evolution during propagation (*35*). The energy flow scenario of a toroidal vortex with longitudinal OAM is similar to that observed for a swirling vortex ring in a rotating fluid. The swirl flow in the toroidal plane reduces the poloidal wavy deformation of the vortex ring and preserves its ring structure. Consequently, the collapse of the ring structure due to the poloidal wavy deformation as seen in vortex rings without swirl does not appear (*36*). Then, the traveling distance of a vortex ring can be extended using the swirl flow under certain conditions. While the presence of a swirl component of velocity makes the edge of the ring roll up, splitting and peeling off, thus preventing the vortex line from forming (*37*–*39*). This paper also considers distinct forms of energy flow associated with toroidal vortices, characterized as saddle-like in normal dispersion and “parallel” in vacuum. Although such structures are difficult to realize in fluidic systems, they are of considerable interest in optics owing to their distinctive propagation dynamics and field configurations, including phenomena such as vortex line reconstruction and robust wave packet transport.

## MATERIALS AND METHODS

### Precise dispersion control via programmable group velocity delay management

In the experiment, the input Gaussian pulsed laser beam is emitted from a laboratory-built source, with a spatial 1/e radius of approximately 2 mm and a spectral bandwidth of ~20 nm centered at 1012 nm (section S5). The pulse is incident at an angle of α=46° onto a reflective blazed grating with a groove density of N=1200 lines/mm. The first-order diffraction (m=+1) disperses the beam horizontally, providing a spectral angular resolution of ∆α/∆λ=mN/cosα=0.0799°/nm. The dispersed beam is subsequently collimated by a cylindrical lens with a focal length of f=100 mm and projected onto an SLM with a pixel pitch of 3.74 μm, covering a spatial extent of ∆L=2ftan∆α/2=2.8mm. This setup yields a frequency-to-pixel mapping of $\omega \left(x\right)=4.76\times 10^{-5}\;\text{rad}/\text{fs}\;\text{per}\;\text{pixel}$ on the SLM. The angular frequency corresponding to the central wavelength is identified by introducing a 0–π phase step at $x=x_{0}$. Consequently, a direct relationship between the applied phase modulation ψ and the induced group delay dispersion is established, given by $\psi =\text{exp}\left\{i\text{GDD}\left[\omega \left(x\right)x-x_{0}\right]\right\}.$ Furthermore, to suppress residual modulation, the phase-only SLM must be calibrated to achieve a linear 2π phase response across all 256 gray levels at the center wavelength.

## Supplementary Materials

### The PDF file includes:

Legends for movies S1 and S2 Sections S1 to S7 Figs. S1 to S6 References
