# Supplementary material for: Dynamics of photonic toroidal vortices mediated by orbital angular momenta
Source: Sci Adv. 2025 Sep 26;11(39):eadz0843. doi: 10.1126/sciadv.adz0843 (PMC13155553; doi:10.1126/sciadv.adz0843)
Supplement: Supplementary file 1 — Legends for movies S1 and S2 Sections S1 to S7 Figs. S1 to S6 References [file sciadv.adz0843_sm.pdf]

Science Advances
AAAS

Supplementary Materials for

Dynamics of photonic toroidal vortices mediated by orbital angular momenta

Xin Liu *et al.*

Corresponding author: Xin Liu, cnliuxin1995@gmail.com; Chunhao Liang, cliang@dal.ca;
Qiwen Zhan, qwzhan@usst.edu.cn; Yangjian Cai, yangjiancai@sdnu.edu.cn

Sci. Adv. **11**, eadz0843 (2025)
DOI: 10.1126/sciadv.adz0843

The PDF file includes:

Legends for movies S1 and S2
Sections S1 to S7
Figs. S1 to S6
References

Other Supplementary Material for this manuscript includes the following:

Movies S1 and S2

Supplementary Video 1: Numerical results of photonic toroidal vortices with longitudinal OAM $\ell_2 = 10$ propagating over different distances.

Supplementary Video 2: Numerical results of photonic toroidal vortices with various longitudinal OAM at propagation distance $3\mathrm{z_R}$.

Supplementary Section 1: energy density and flow of an STOV pulse with distinct group velocity dispersion

An STOV pulse, whose spatiotemporal envelope at the source (*z*=0) is described by a spatiotemporal Laguerre-Gaussian mode, is given by

$$\Psi(x,z,\tau)=\left(\frac{\sqrt{2}\rho}{w_0}\right)^{|\ell|}\exp\left(-\frac{\rho^2}{w_0^2}\right)L_p^{|\ell|}\left(\frac{2\rho^2}{w_0^2}\right)\exp(-i\ell\varphi), \tag{1}$$

where $\rho = \sqrt{\tau^2/w_\tau^2 + x^2/w_x^2}$, $\varphi = \tan^{-1}(w_\tau x/w_x\tau)$ and w_τ/w_x quantifies the ratio of the width of time to space. w_0 characterizes the beam width. $L_p^{|\ell|}(\cdot)$ denotes associated Laguerre polynomials of the order $p + |\ell|$. Figures S1(A) and S1(B) display the energy density and phase distributions in the space-time plane of a typical STOV with *p*=0 and ℓ=+1 at *z*=0. As described by Eq. (9) and Eq. (10) in Supplementary Section 3, the energy flow is jointly determined by diffraction and dispersion effects. Figures S1 (C)-(E) show the calculated energy density flow $\boldsymbol{J}_{\tau-x}$ in the space-time plane, obtained from Eq. (8) with various β_2. It can be observed that the energy density flow of an STOV pulse with $\beta_2 = k_0^{-1}$ exhibits a “saddle” pattern with respect to the singularity. In this case, the STOV cannot sustain intrinsic transverse OAM (i.e., $\langle L_y \rangle = 0$) and undergoes diagonally splitting during propagation because of a strong spatiotemporal astigmatism, as depicted in Figs. S1(Fa)-(Fc). Beyond a propagation distance approaching the Rayleigh length, the STOV wavepacket exhibits significant degradation, evolving into a multi-lobed structure as illustrated in Fig. S1(Fc). But for an STOV propagates in vacuum with $\beta_2 = 0$, the energy flow is restricted to *x*-axis [see Fig. S1(D)]. In this scenario, the STOV pulse carries a

transverse OAM of $\langle L_y \rangle = \ell/2$ and primarily expands along the x-axis, evolving into a structure characterized by multiple dark regions [Figs. S1(Ga)-(Gc)], exhibiting an overall shape of a tilted Hermite-Gaussian mode[40]. When $\beta_2 = -k_0^{-1}$, the spatial diffraction and temporal dispersion are balanced, the resulting energy flow of an STOV pulse exhibits a "spiral" pattern that circulates around the singularity. This energy flow enables the STOV pulse to retain a well-defined, donut-shaped intensity profile and a preserved helicoid phase structure over a distance [see Figs. S1(Ha)-(Hc)], imparts a transverse OAM of $\langle L_y \rangle = \ell$. As a summary, the cross-section structure of a spatiotemporal wavepacket is directly governed by their energy flow dynamics, which are influenced by the interplay of dispersion and diffraction.

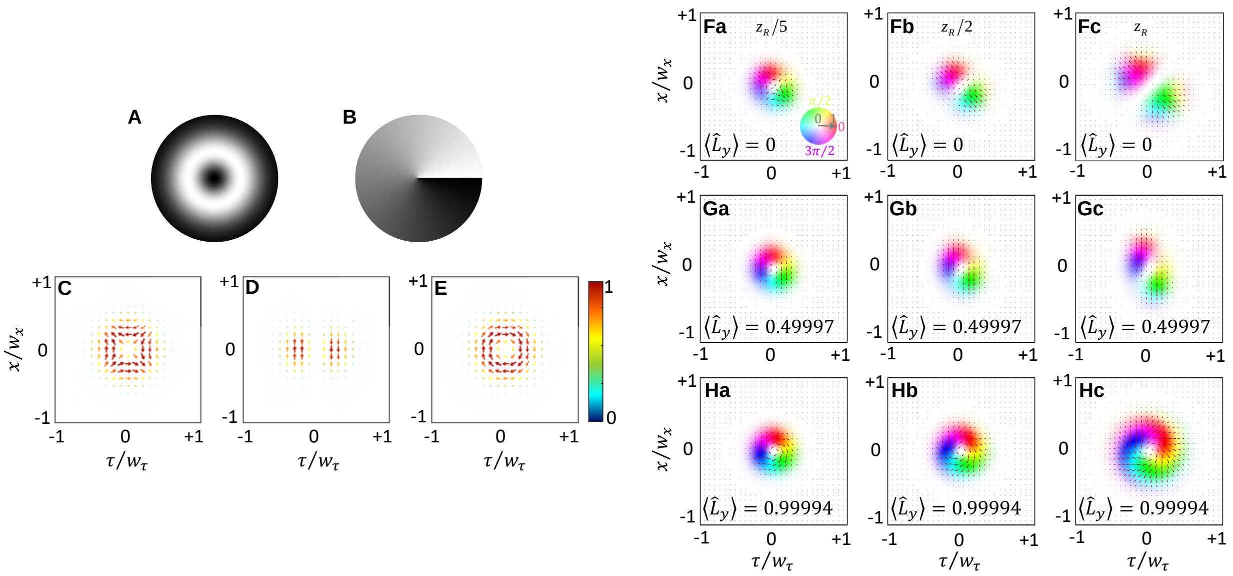

Fig. S1 Analysis of energy density and averaged transverse OAM density of STOV wavepackets. Energy density (**A**) and phase (**B**) distribution of an STOV (ℓ=+1) wavepacket (defined in the space-time plane). (**C**)-(**E**) Calculated energy density flow $\mathbf{J}_{\tau-x}$ of an STOV wavepacket with (**C**) $\beta_2 = k_0^{-1}$, (**D**) $\beta_2 = 0$ and (**E**) $\beta_2 = -k_0^{-1}$. (**F**)-(**G**) Complex field of an STOV (ℓ=+1) wavepacket at different propagation distances with (**Fa**)-(**Fc**): $\beta_2 = k_0^{-1}$; (**Ga**)-(**Gc**): $\beta_2 = 0$ and (**Ha**)-(**Hc**) $\beta_2 = -k_0^{-1}$. The propagation distances from left to right are $0.2z_R$,$0.5z_R$ and z_R. In each plot, the saturation and hue denote energy density and phase distribution

respectively. The energy flow in the local time frame is indicated by the red arrows. The average transverse OAM values $\langle L_y \rangle$ of an STOV pulse at different propagation distances are calculated using Eq. (11). The parameters are $\lambda_0 = 800$nm w_τ =1ps, $w_x = 1$mm, w_0 =0.25.

Supplementary Section 2: energy flow of toroidal vortices

Figure S2 illustrates the calculated energy flow of toroidal wavepackets of $\ell_2 = 0$ with various β_2. In Fig. S2(A), a toroidal vortex lacking longitudinal OAM exhibits an energy flow in the poloidal plane identical to that of an STOV pulse. However, in Fig. S2(B), the toroidal vortex with longitudinal OAM, possessing both transverse [$\langle \hat{L}_x \rangle$ and $\langle \hat{L}_y \rangle$ in the poloidal plane] and longitudinal [$\langle \hat{L}_\tau \rangle \propto \ell_2 \neq 0$ in the toroidal plane] OAM, displays an energy density flow with a distinct toroidal component. We would like to mention that, as described in the main text, the energy flow patterns of photonic toroidal vortices with or without longitudinal OAM in anomalous dispersion closely resemble those of vortex rings with or without swirl in fluid dynamics, exhibiting analogous motion and dynamic behavior[36-40].

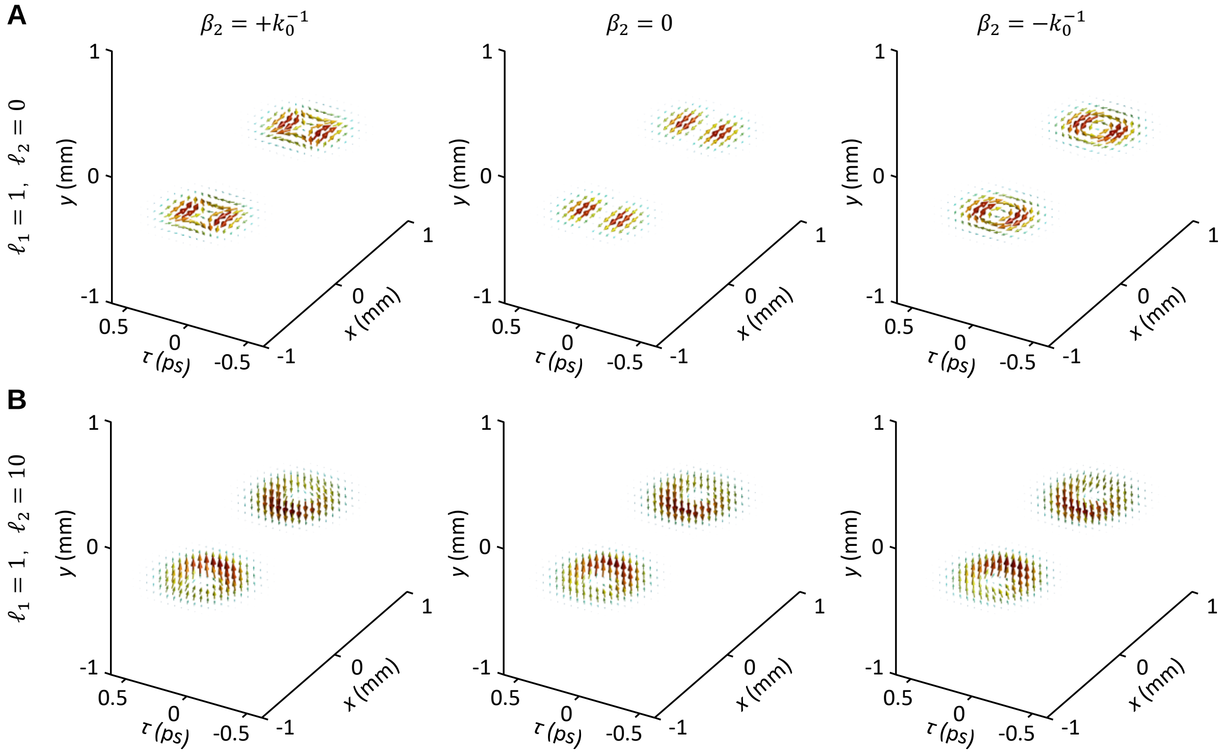

Fig. S2 Analysis of energy density flow in photonic toroidal vortices. Calculated energy density flow [Eq. (8)] of a photonic toroidal vortex (**A**) without or (**B**) with longitudinal OAM under various β_2. Take a specific poloidal plane (y=0) as an example. Other parameters are the same as in Fig. 1 of the main text.

Supplementary Section 3: numerical calculation and momentum flow of propagation for a full space-time wavepacket in dispersive media

Based on the electromagnetic theory of light and Maxwell's equations, the propagation of a light pulse, characterized by a scalar complex field $E(x,y,z;t)$ of center carrier frequency ω_0, in an isotropic, transparent, passive linear medium is governed by the following wave equation

$$\left(\frac{\partial^2}{\partial x^2}+\frac{\partial^2}{\partial y^2}+\frac{\partial^2}{\partial z^2}\right)E(x,y,z;t)-\frac{1}{c^2}\frac{\partial^2 E(x,y,z;t)}{\partial t^2}=0, \tag{2}$$

where c is the speed of light in vacuum. We assume that the light pulse is travelling at both paraxial approximation with $|\Delta k| \ll k_0$ and narrow bandwidth approximation with $|\Delta\omega| \ll \omega_0$. With these approximations, the froward (z>0) electric field can be expressed as the product of a slowly varying envelop and the carrier oscillation at the central frequency, given by $E(x,y,z;t) = \Psi(x,y,z;t)\exp(-i\omega_0 t + ik_0 z)$, where $k_0 = \omega_0/c = 2\pi/\lambda_0$ is a propagation constant. In the temporal frequency domain, the electric field has $\hat{E}(x,y,z;\omega) = \hat{\Psi}(x,y,z;\Omega)\exp(ik_0 z)$ with the detuning angular frequency $\Omega = \omega - \omega_0$, where the top mark denotes time-only Fourier transform: $\hat{f}(\omega) = \int f(t)\exp(-i\omega t)\,dt$. Taking the replacing operators $\partial E/\partial t = -i\omega_0 \hat{E}$ and $\partial^2 E/\partial t^2 = -\omega_0^2 \hat{E}$, one can obtain the paraxial wave equation in the frequency form

$$\left(\frac{\partial^2}{\partial x^2}+\frac{\partial^2}{\partial y^2}\right)\hat{\Psi}+2ik_0\frac{\partial\hat{\Psi}}{\partial z}+\left[k^2(\omega)-k_0^2\right]\hat{\Psi}=0. \tag{3}$$

It should be noted that the derivation of Eq. (3) from Eq. (2) relies on two slowly varying envelope approximations (SVEAs): (i) spatial SVEA, the distance light travels during the duration of a pulse is much larger than its wavelength, i.e.,$|\partial^2 E/\partial z^2| \ll |k\partial E/\partial z| \ll |k^2 E|$, and (ii) temporal SVEA,

the temporal width of the optical envelop is much larger than its carrier period, i.e.,$|\partial^2 E/\partial t^2| \ll |\omega \partial E/\partial t| \ll |\omega^2 E|$. As a result, the second-order derivative $\partial^2 E/\partial z^2$is negligible with respect to $k\partial E/\partial z$ during the above derivation. In Eq. (3), the paraxial approximation allows $k^2(\omega) - k_0^2 \approx 2k_0[k(\omega) - k_0]$ and the Taylor expands of $k(\omega)$ reads $k(\omega) = \sum_{m=0}^{+\infty} k^{(m)}(\omega_0)\Omega^m/m!$, where $v_g = 1/k^{(1)}(\omega_0)$ and $\beta_2 = k^{(2)}(\omega_0)$ is group velocity and group velocity dispersion (GVD) at ω_0, respectively. By expanding the first three terms in a Taylor series and applying the inverse operators $-i\Omega\widetilde{\Psi} = \partial\Psi/\partial t$, $-\Omega^2\widetilde{\Psi} = \partial^2\Psi/\partial t^2$, we can readily obtain

$$\frac{\partial \Psi}{\partial z} = \frac{i}{2k_0}\left(\frac{\partial^2}{\partial x^2} + \frac{\partial^2}{\partial y^2}\right)\Psi - i\frac{\beta_2}{2}\frac{\partial^2 \Psi}{\partial \tau^2}, \tag{4}$$

where $\tau = t - z/v_g$ is the local variables. A solution to Eq. (4) is given by:

$$\Psi(x,y,z,\tau) = \frac{\exp\left[ik_0 z + ik_0 (x^2+y^2)/2z\right]}{2\pi} \times \iiint \Psi(x_0, y_0, 0, \Omega) \\ \exp\left(ik_0 \frac{x_0^2 + y_0^2}{2z} + i\beta_2 \frac{\Omega^2}{2} z\right) \exp\left(-ik_0 \frac{xx_0 + yy_0}{z} - i\Omega\tau\right) d\Omega dx_0 dy_0, \tag{5}$$

and an angular spectrum relation

$$\Psi(x,y,z,\tau) = \frac{\exp(ik_0 z)}{8\pi^3} \times \\ \iiint \tilde{\Psi}(k_x, k_y, \Omega) H(k_x, k_y, \Omega) \exp(-ik_x x - ik_y y - i\Omega\tau) d\Omega dk_x dk_y, \tag{6}$$

where $H(k_x, k_y, \Omega) = \exp\left[-i\left(k_x^2 + k_y^2\right)z/2k_0 + i\beta_2\, \Omega^2 z/2\right]$. For a full space-time optical wavepacket with entangled 3D variables, obtaining closed-form solutions for Eqs. (5) and (6) is challenging. The above integral equations are numerically obtainable using the Fast Fourier Transform algorithm. For a scalar wavepacket $\Psi(x, y, \varsigma; \tau)$, the square of its modulus $|\Psi|^2$ represents the energy density at a given position. Integrating over a volume *V* in the *x*-*y*-*τ* space $\int_V |\Psi|^2 dx dy d\tau$, gives the total energy carried by the wavepacket. The total energy satisfies an

energy conservation equation, which can be derived by multiplying Eq. (4) by the complex conjugate of the wavepacket. This equation is expressed as:

$$\begin{aligned}\frac{\partial|\Psi|^2}{\partial\varsigma}=&-\frac{i}{2k_0}\left[\frac{\partial}{\partial x}\left(\Psi\frac{\partial\Psi^*}{\partial x}-\Psi^*\frac{\partial\Psi}{\partial x}\right)+\frac{\partial}{\partial y}\left(\Psi\frac{\partial\Psi^*}{\partial y}-\Psi^*\frac{\partial\Psi}{\partial y}\right)\right]\\&-i\frac{\beta_2}{2}\frac{\partial}{\partial\tau}\left(\Psi^*\frac{\partial\Psi}{\partial\tau}-\Psi\frac{\partial\Psi^*}{\partial\tau}\right).\end{aligned}\tag{7}$$

The right-hand side of Eq. (7) takes an analogous form to the definition of the divergence operator in the *x*-*y*-*τ* space[41], as follows

$$\frac{\partial|\Psi|^2}{\partial\varsigma}=-\mathrm{div}\mathbf{J}=-\nabla_\perp\cdot\mathbf{J}_\perp-\frac{\partial\mathbf{J}_\tau}{\partial\tau},\tag{8}$$

where $\nabla_\perp=\partial/\partial x\,\vec{\boldsymbol{x}}+\partial/\partial y\vec{\boldsymbol{y}}$ denotes the transverse curl operator. Equation (8) enables the identification of the total energy density flux $\mathbf{J}=\mathbf{J}_\perp+\mathbf{J}_\tau$ through the whole surface of an enclosing volume *V*, is given by $\mathbf{J}_\perp=\frac{i}{2k_0}(\Psi^*\nabla_\perp\Psi-\Psi\nabla_\perp\Psi^*)$ and $\mathbf{J}_\tau=i\frac{\beta_2}{2}\left(\Psi^*\frac{\partial\Psi}{\partial\tau}-\Psi\frac{\partial\Psi^*}{\partial\tau}\right)\vec{\boldsymbol{\tau}}$.

The cross product of the energy density flow $\mathbf{J}$ with position vector $\vec{\mathbf{r}}=x\vec{\mathbf{x}}+y\vec{\mathbf{y}}+\tau\vec{\boldsymbol{\tau}}$ gives an OAM operator below

$$\begin{aligned}\hat{L}_x&=-\frac{i}{k_0}\left(-yk_0\beta_2\frac{\partial}{\partial\tau}-\tau\frac{\partial}{\partial y}\right),\\\hat{L}_y&=-\frac{i}{k_0}\left(\tau\frac{\partial}{\partial x}+xk_0\beta_2\frac{\partial}{\partial\tau}\right),\\\hat{L}_\tau&=-\frac{i}{k_0}\left(x\frac{\partial}{\partial y}-y\frac{\partial}{\partial x}\right).\end{aligned}\tag{9}$$

Equation (9) can also be expressed in the cylindrical coordinate as follows:

$$
\begin{aligned}
\hat{L}_x &= -\frac{i}{k_0}\left[-(1+k_0\beta_2)\frac{\partial}{\partial r}r\cos\theta\sin\theta+\left(k_0\beta_2\sin^2\theta-\cos^2\theta\right)\frac{\partial}{\partial\theta}\right],\\
\hat{L}_y &= -\frac{i}{k_0}\left[(1+k_0\beta_2)\frac{\partial}{\partial\rho}\rho\sin\varphi\cos\varphi+\left(\cos^2\varphi-k_0\beta_2\sin^2\varphi\right)\frac{\partial}{\partial\varphi}\right],\\
\hat{L}_\tau &= -\frac{i}{k_0}\frac{\partial}{\partial\gamma}\vec{\boldsymbol{\tau}},
\end{aligned}
\tag{10}
$$

where $r=\sqrt{\tau^2+y^2}, \theta=\tan^{-1}(y/\tau)$, $\rho=\sqrt{\tau^2+x^2}, \varphi=\tan^{-1}(x/\tau)$ and $\gamma=\tan^{-1}(y/x)$. Equation (10) reveals that the first term of each expression represents extrinsic transverse OAM, while the second term represents intrinsic transverse OAM per photon, determined by not only the phase gradient but also the GVD coefficient. Equation (10) describes the longitudinal OAM $\hat{L}_\tau$, which is orthogonal to the transverse OAM characterized by $\hat{L}_x$ and $\hat{L}_y$. The standard definition of the average T-OAM is expressed as[25,42]

$$
\langle L_y\rangle=\hbar\frac{\langle\Psi|\hat{L}_y|\Psi\rangle}{\langle\Psi|\Psi\rangle}=\hbar\frac{\iint\Psi^*|\hat{L}_y\Psi d\tau dx}{\iint\Psi^*\Psi d\tau dx},
\tag{11}
$$

where $\hbar$ is a Planck constant divided by 2π, and $\hat{L}_y$, $\hat{L}_x$ and $\hat{L}_\tau$ denote OAM operators as defined in Eq. (9).

Supplementary Section 4: the rule for balancing the poloidal diffraction and toroidal diffraction

In our study, the coupling of temporal dispersion and spatial diffraction gives rise to transverse OAM (ℓ_1) and the associated energy flow in the poloidal plane, while the two-dimensional spatial diffraction contributes to longitudinal OAM (ℓ_1) and generates a swirling energy flow in the toroidal plane. To ensure comparable diffraction effects in both the poloidal and toroidal planes, the corresponding Rayleigh lengths should be matched, i.e., $z_{Rp}\sim z_{Rt}$, leading to an equivalent diffraction condition $w_0\sim w_s$. The toroidal beam width w_s can be approximately determined from the relationship between the radius r_0 (defined as the distance from the beam center to the point of maximum intensity) and the width w_s of a Laguerre-Gaussian mode with radial index

$p = 0$, given by $r_0 = w_s\sqrt{|\ell_2|/2}$. This yields the approximate relation $r_0 \sim w_0\sqrt{|\ell_2|/2}$, which serves as a basis for ruling the propagation dynamics of toroidal vortices mediated by the interplay between transverse and longitudinal OAM, jointly governed by dispersion and diffraction. A significant interaction between the transverse and longitudinal OAM occurs only when this rule is satisfied.

Supplementary Section 5: input pulse and toroidal vortex characterization

In the experiment, the ultrafast laser is a dispersion-managed mode-locked fiber laser. Figure S3 (A) shows the spectrum of the laser center at 1012nm with a bandwidth of ~20nm. The duration of the generated photonic toroidal vortices, exceeding 1 picosecond, is longer than that of the reference pulse. The complete space-time structure can be reconstructed from a series of time-delayed interference fringes, produced via off-axis (~0.25 degree) interference with a transform-limited reference pulse, as shown in Figs. S3(B) and S3(C).

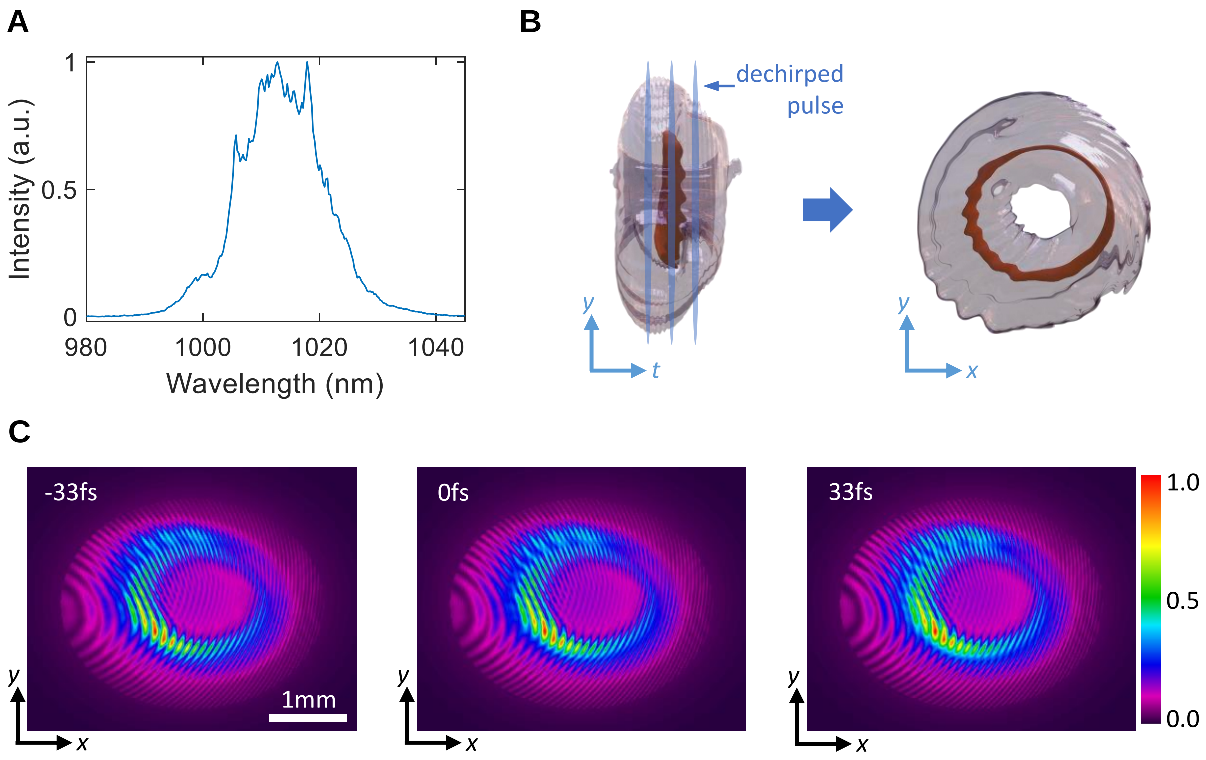

Fig. S3 Characterization of input pulsed beam and toroidal vortex measurement. (**A**) Spectrum of the fiber laser. (**B**) Characterization of spatiotemporal toroidal vortex. (**C**) Some representative interference patterns of the generated toroidal vortex of $\ell_1 = 1$ and $\ell_2 = 10$ with the dechirped pulsed beam at different times.

Supplementary Section 6: Evolutions of the iso-intensity surfaces of propagated toroidal vortices with different ℓ_2 and different distances

In this section, we investigate the effect of topological charge ℓ_2 values on the propagation dynamics of toroidal vortices. Figure S4 presents the experimentally measured iso-intensity surfaces of toroidal vortices at a propagation distance of $3z_R$ with increasing ℓ_2, illustrating the detailed evolution process shown in Fig. 6 of the main text. The corresponding simulated results are provided in Supplementary Video 2. It is observed that under normal [Fig. S4(A)] and anomalous dispersion [Fig. S4(B)], the toroidal vortices undergo temporal splitting, but in opposite directions. In contrast, in vacuum [i.e., without dispersion, Fig. S4(C)], the morphology of the toroidal vortices and the associated vortex line are progressively recovered with increasing ℓ_2, ultimately forming a robust structure as depicted in Fig. 6 of the main text. The experimental results are in good agreement with the simulations.

Figure S5 presents the dynamics of photonic toroidal vortices with $\ell_2 = 10$ over various propagation distances, corresponding to Fig. 6 of the main text. Under both normal [Fig. S5(A)] and anomalous [Fig. S5(B)] dispersion conditions, the vortex structure becomes unstable during propagation, gradually splitting along the temporal axis and eventually evolving into a pair of toroidal rings with opposite orientations. While in vacuum [Fig. S5(C)] , the initially fragmented photonic toroidal vortex progressively recovers its toroidal form and reconnects its vortex line during propagation. These experimental observations are in good agreement with the simulation

results and highlight the nontrivial dynamics of photonic toroidal vortices mediated by the interplay between transverse and longitudinal OAM.

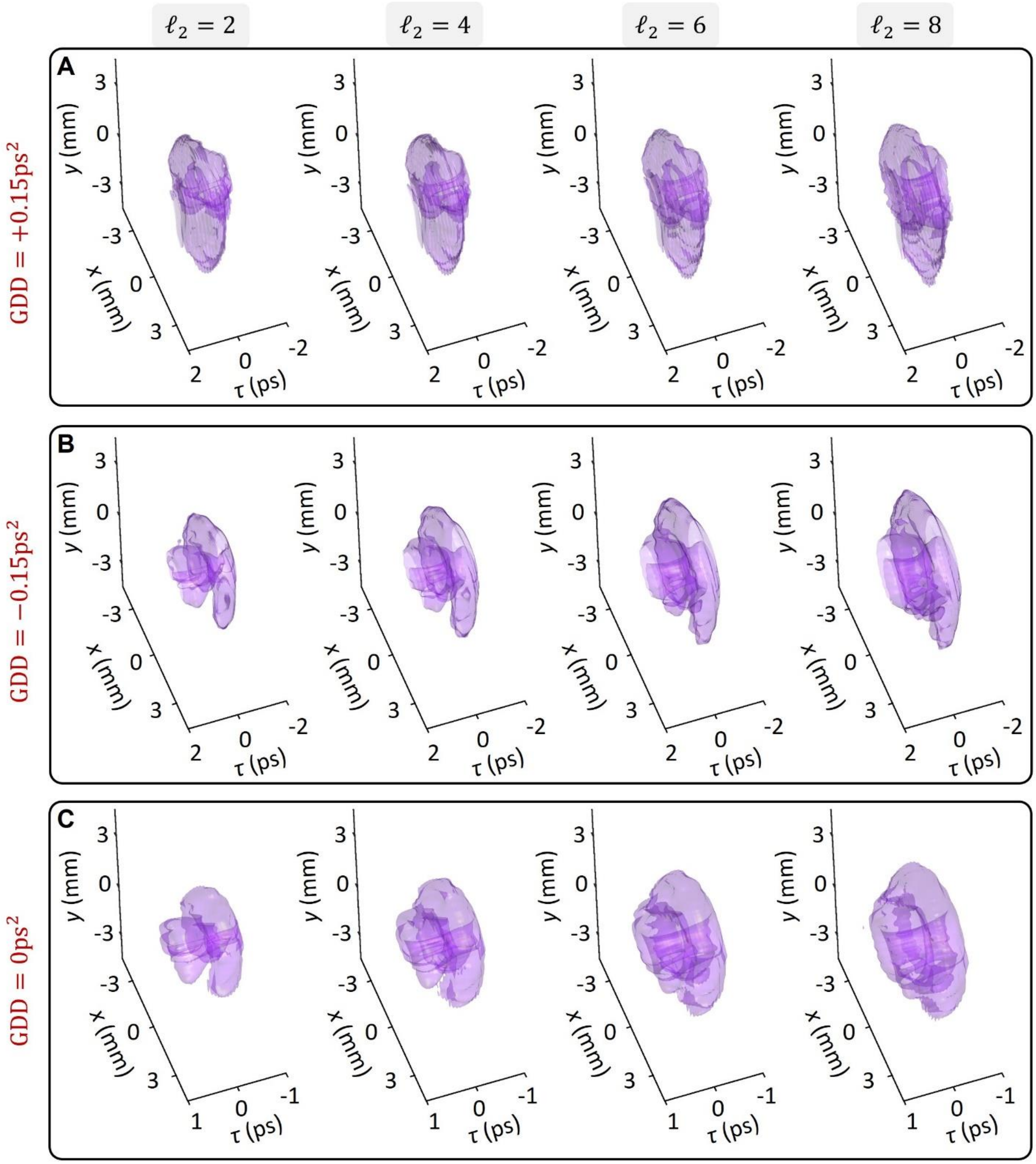

Fig. S4 3D iso-intensity surfaces of toroidal vortices with various ℓ_2 values, measured at a propagation distance of 0.8m, approximately 3z_R. (**A**) normal dispersion (GDD=+0.15ps^2),

corresponding to Fig. 6(D); (**B**) anomalous dispersion (GDD=-0.15ps^2), corresponding to Fig. 6(E); (**C**) vacuum (without GDD), corresponding to Fig. 6(C). The iso-values are set to 1% of the peak intensity.

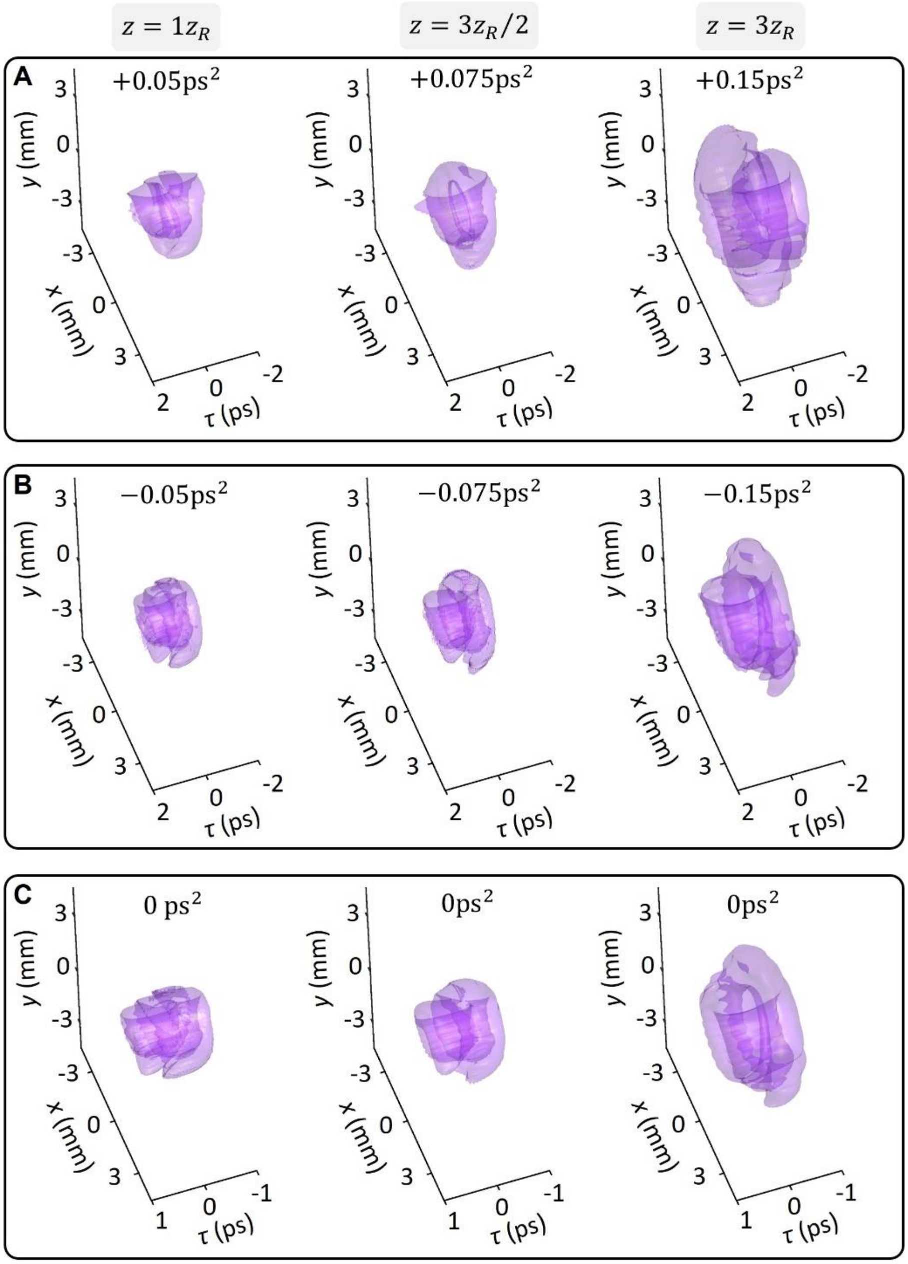

Fig. S5 3D iso-intensity surfaces of toroidal vortices with $\ell_2 = 10$, measured at different propagation distances. (**A**) normal dispersion, corresponding to Fig. 6(D); (**B**) anomalous

dispersion, corresponding to Fig. 6(E); (**C**) vacuum, corresponding to Fig. 6(C). The iso-values are set to 1% of the peak intensity.

Supplementary Section 7: numerical analysis of photonic toroidal vortex dynamics using fidelity

The propagated photonic toroidal vortex exhibits a ring-shaped, circularly symmetric structure in the toroidal plane and ideally maintains a circularly symmetric spatiotemporal vortex in the poloidal plane [Fig. S6(A)]. Therefore, the fidelity F, defined as the energy overlap integral between the poloidal structure $\Psi_{\mathrm{PTV}}(\tau, x)$ of the propagated toroidal vortex and that of an ideal spatiotemporal vortex $\Psi_{\mathrm{STV}}(\tau, x)$, can be used to evaluate the degree of structural preservation, and is given by

$$\mathbf{F} = \frac{\iint \left|\Psi_{\mathrm{PTV}}(\tau, x)\right| \cdot \left|\Psi_{\mathrm{STV}}(\tau, x)\right| d\tau dx}{\left[\iint \left|\Psi_{\mathrm{PTV}}(\tau, x)\right| d\tau dx \cdot \iint \left|\Psi_{\mathrm{STV}}(\tau, x)\right| d\tau dx\right]^{1/2}}. \tag{12}$$

To match the dimension between poloidal vortex and theoretical vortex, the coordinates of space-time plane need to be scaled to obtain the fidelity correctly, by utilizing the method adopted in [42,43]. we first normalize the original coordinate (τ, x) to a normalized coordinate $(\tau_{\mathrm{norm}}, x_{\mathrm{norm}})$, where $\tau_{\mathrm{norm}} = (\tau - \tau_0)/2t_0$ and $x_{\mathrm{norm}} = (x - x_0)/2w_0$. (τ_0, x_0) is the central position of the poloidal vortex. We designate this center point as the origins and then proceed to draw cross lines along the time and space directions from these origins, resulting in two pairs of intensity peaks respectively. We then define the normalized widths t_0 and w_0 to be one-half of the peak-to-peak separations along the time and space directions, respectively. Finally, the corresponding theoretical vortex can be correctly expressed as $\Psi_{\mathrm{STV}}(\tau, x) \propto \sqrt{2}(x_{\mathrm{norm}} + i\tau_{\mathrm{norm}})e^{-2(x_{\mathrm{norm}}^2 + \tau_{\mathrm{norm}}^2)}$. Figures S6(B) and S6(C) show the poloidal vortex and theoretical vortex depicted in the normalized coordinate, in which they have identical scales in both space and time. The calculated fidelity curves of the propagated toroidal vortex at $3z_R$ as a function of ℓ_2 are

plotted in Fig. S6(D). It can be seen that with increasing longitudinal OAM, the propagated toroidal vortex undergoes significant reconstruction in vacuum, while it degrades under both normal and anomalous dispersion. The fidelity in vacuum is clearly higher than in dispersive media. A comparison between Figs. S6(Da) and S6(Db) further reveal that a well-defined central dark region is reformed in vacuum, indicating the re-establishment of the vortex line within the toroidal structure. As propagation continues [Fig. S6(E)], the fidelity in vacuum increases and becomes nearly independent of ℓ_2, indicating that the renascent toroidal vortex reaches a stable state. In contrast, the toroidal vortex propagating with dispersion undergoes significant deformation, as illustrated in Figs. S6(Ea) and S6(Eb). All of these quantitative analyses are consistent with the conclusions presented in the main text.

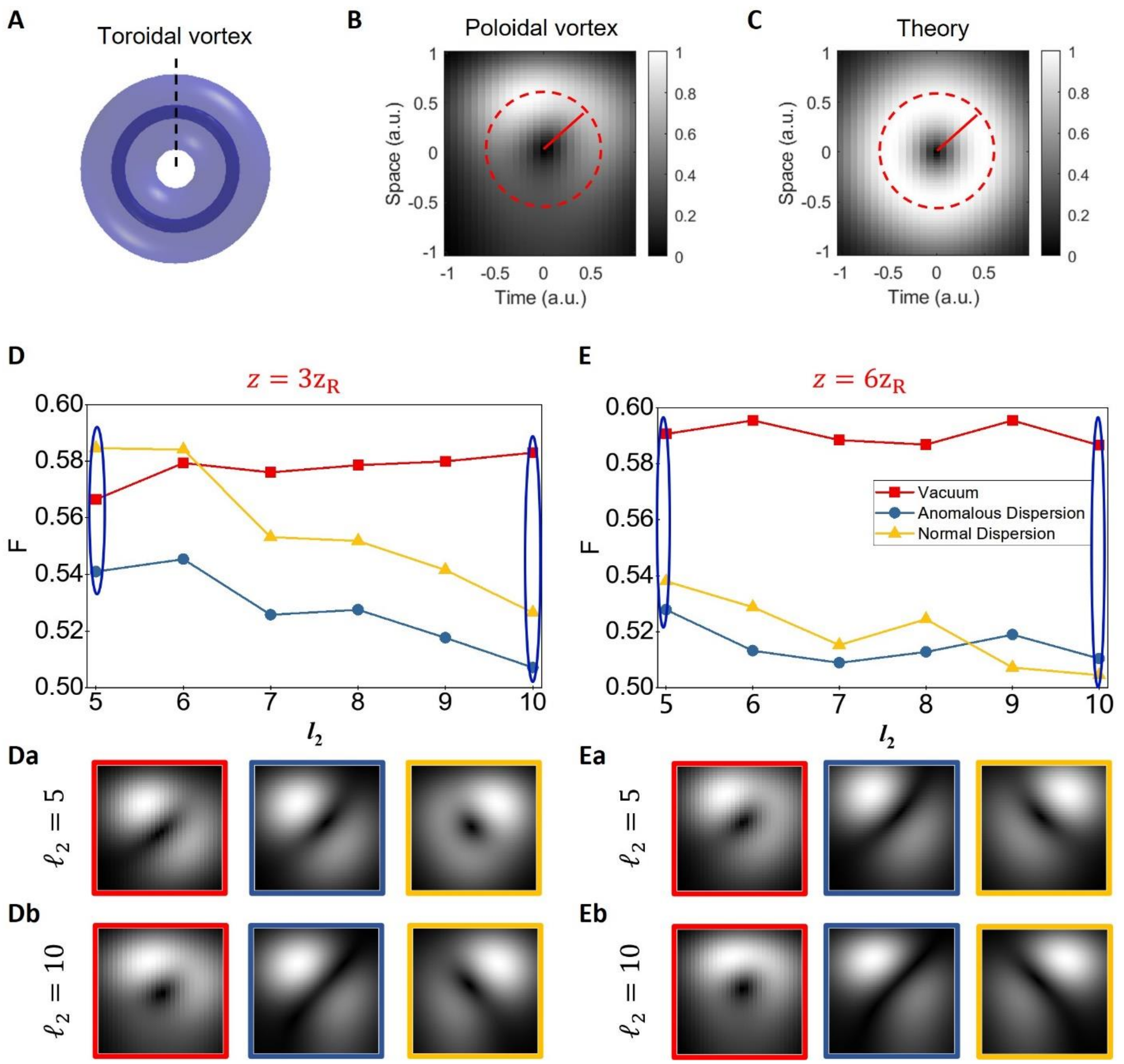

Fig. S6 Fidelity of the propagated toroidal vortex as a function of ℓ_2 at different distances. (**A**) Toroidal vortex consists of a closed STOV tube. (**B**) Vortex profile at a specific poloidal plane. (**C**) Intensity of a theoretical STOV. (**D**) Fidelity as a function of longitudinal OAM values across different dispersion conditions at a propagation distance $z = 3z_R$. (**Da**) Poloidal vortex profiles under various dispersion conditions for $\ell_2 = 5$. (**Db**) Poloidal vortex profiles under various dispersion conditions for $\ell_2 = 10$. (**E**) Fidelity as a function of longitudinal OAM values across different dispersion conditions at a propagation distance $z = 6z_R$. (**Ea**) Poloidal vortex profiles

under various dispersion conditions for $\ell_2 = 5$. (**Eb**) Poloidal vortex profiles under various dispersion conditions for $\ell_2 = 10$. All parameters are consistent with those used in Figs. 2-4 of the main text.

REFERENCES AND NOTES

1. D. G. Akhmetov, *Vortex Rings* (Springer Science & Business Media, 2009).

2. T. Matsuzawa, N. P. Mitchell, S. Perrard, W. T. M. Irvine, Creation of an isolated turbulent blob fed by vortex rings. *Nat. Phys.* **19**, 1193–1200 (2023).

3. J. S. Lee, S. J. Park, J. H. Lee, B. M. Weon, K. Fezzaa, J. H. Je, Origin and dynamics of vortex rings in drop splashing. *Nat. Commun.* **6**, 8187 (2015).

4. T. T. Lim, T. B. Nickels, Vortex Rings. *Fluid Vortices* **30**, 95–153 (1995).

5. S. Hess, L. Eme, A. J. Roger, A. G. B. Simpson, A natural toroidal microswimmer with a rotary eukaryotic flagellum. *Nat. Microbiol.* **4**, 1620–1626 (2019).

6. D. L. Whitaker, J. Edwards, Sphagnum moss disperses spores with vortex rings. *Science* **329**, 406–406 (2010).

7. C. Cummins, M. Seale, A. Macente, D. Certini, E. Mastropaolo, I. M. Viola, N. Nakayama, A separated vortex ring underlies the flight of the dandelion. *Nature* **562**, 414–418 (2018).

8. P. J. Kilner, G.-Z. Yang, A. J. Wilkes, R. H. Mohiaddin, D. N. Firmin, M. H. Yacoub, Asymmetric redirection of flow through the heart. *Nature* **404**, 759–761 (2000).

9. H. Aref, I. Zawadzki, "Linking of vortex rings. *Nature* **354**, 50–53 (1991).

10. P. Chatelain, D. Kivotides, A. Leonard, Reconnection of colliding vortex rings. *Phys. Rev. Lett.* **90**, 054501 (2003).

11. T. T. Lim, T. B. Nickels, Instability and reconnection in the head-on collision of two vortex rings. *Nature* **357**, 225–227 (1992).

12. S. Zhao, J. Tao, Instability of a rotating liquid ring. *Phys. Rev. E* **88**, 033016 (2013).

13. I. S. Sullivan, J. J. Niemela, R. E. Hershberger, D. Bolster, R. J. Donnelly, Dynamics of thin vortex rings. *J. Fluid Mech.* **609**, 319–347 (2008).

14. A. Zdagkas, C. McDonnell, J. Deng, Y. Shen, G. Li, T. Ellenbogen, N. Papasimakis, N. I. Zheludev, Observation of toroidal pulses of light. *Nat. Photonics* **16**, 523–528 (2022).

15. Y. Shen, Y. Hou, N. Papasimakis, N. I. Zheludev, Supertoroidal light pulses as electromagnetic skyrmions propagating in free space. *Nat. Commun.* **12**, 5891 (2021).

16. R. Wang, B. Ying, S. Shi, J. Wang, B. Z. Wang, M. Liang, Y. Shen, Hybrid electromagnetic toroidal vortices. *Sci. Adv.* **11**, eads4797 (2025).

17. R. Wang, P.-Y. Bao, X. Feng, J. Wu, B. Z. Wang, Y. Shen, Single-antenna super-resolution positioning with nonseparable toroidal pulses. *Commun. Phys.* **7**, 356 (2024).

18. Y. Shen, Q. Zhang, P. Shi, L. Du, X. Yuan, A. V. Zayats, Optical skyrmions and other topological quasiparticles of light. *Nat. Photonics* **18**, 15–25 (2024).

19. Y. Shen, H. Wang, S. Fan, Free-space topological optical textures: Tutorial. *Adv. Opt. Photonics* **17**, 295–374 (2025).

20. Y. Shen, N. Papasimakis, N. I. Zheludev, Nondiffracting supertoroidal pulses: Optical "Kármán vortex streets." *Nat. Commun.* **15**, 4863 (2024).

21. R. Wang, P.-Y. Bao, Z.-Q. Hu, S. Shi, B. Z. Wang, N. I. Zheludev, Y. Shen, Observation of resilient propagation and free-space skyrmions in toroidal electromagnetic pulses. *Appl. Phys. Rev.* **11**, 031411 (2024).

22. R. Wang, D.-T. Yang, T. Xin, S. Shi, B. Z. Wang, Y. Shen, Optical atompilz: Propagation-invariant strongly longitudinally polarized toroidal pulses. *Appl. Phys. Lett.* **125**, 111101 (2024).

23. C. Wan, Q. Cao, J. Chen, A. Chong, Q. Zhan, Toroidal vortices of light. *Nat. Photonics* **16**, 519–522 (2022).

24. S. W. Hancock, S. Zahedpour, A. Goffin, H. M. Milchberg, Free-space propagation of spatiotemporal optical vortices. *Optica* **6**, 1547–1553 (2019).

25. A. Chong, C. Wan, J. Chen, Q. Zhan. Generation of spatiotemporal optical vortices with controllable transverse orbital angular momentum. *Nat. Photonics* **14**, 350–354 (2020).

26. X. Liu, Q. Cao, N. Zhang, A. Chong, Y. Cai, Q. Zhan, Spatiotemporal optical vortices with controllable radial and azimuthal quantum numbers. *Nat. Commun.* **15**, 5435 (2024).

27. Q. Zhan, Spatiotemporal sculpturing of light: A tutorial. *Adv. Opt. Photonics* **16**, 163 (2024).

28. X. Liu, Q. Cao, Q. Zhan, Spatiotemporal optical wavepackets: From concepts to applications. *Photon. Insights* **3**, R08 (2024).

29. W. Chen, Y. Liu, A. Yu, H. Cao, W. Hu, W. Qiao, L. S. Chen, Y. Q. Lu, Observation of chiral symmetry breaking in toroidal vortices of light. *Phys. Rev. Lett.* **132**, 153801 (2024).

30. C. Wan,Y. Shen, A. Chong, Q. Zhan, Scalar optical hopfions. *eLight* **2**, 22 (2022).

31. Z. Lyu, Y. Fang, Y. Liu, Formation and controlling of optical hopfions in high harmonic generation. *Phys. Rev. Lett.* **133**, 133801 (2024).

32. Q. Cao, N. Zhang, A. Chong, Q. Zhan, Spatiotemporal photonic emulator of potential-free Schrödinger equation.. *eLight* **5**, 17 (2025).

33. Q. Cao, J. Chen, K. Lu, C. Wan, A. Chong, Q. Zhan, Non-spreading Bessel spatiotemporal optical vortices. *Sci. Bull.* **67**,133–140 (2022).

34. W. Chen, W. Zhang, Y. Liu, F. C. Meng, J. M. Dudley, Y. Q. Lu, Time diffraction-free transverse orbital angular momentum beams. *Nat. Commun.* **13**, 4021 (2022).

35. P. S. Krueger, M. Gharib, The significance of vortex ring formation to the impulse and thrust of a starting jet. *Phys. Fluids* **15**, 1271–1281 (2003).

36. T. Naitoh, N. Okura, T. Gotoh, Y. Kato, On the evolution of vortex rings with swirl. *Phys. Fluids*, **26**, 067101 (2014).

37. R. Verzicco, P. Orlandi, A. H. M. Eisenga, A. H. M. Eisenga, G. J. F. Van Heijst, G. F. Carnevale, Dynamics of a vortex ring in a rotating fluid. *J. Fluid Mech.* **317**, 215–239 (1996).

38. C. Gargan-Shingles, M. Rudman, K. Ryan, The evolution of swirling axisymmetric vortex rings. *Phys. Fluids* **27**, 087101 (2015).

39. D. Virk, M. V. Melander and F. Hussain, Dynamics of a polarized vortex ring. *J. Fluid Mech.* **260**, 23–55 (1994).

40. M. A. Porras, Propagation of higher-order spatiotemporal vortices. *Opt. Lett.* **48**, 367–370 (2023).

41. A. Lotti, A. Couairon, D. Faccio, P. D. Trapani, Energy-flux characterization of conical and space-time coupled wave packets. *Phys. Rev. A* **81**, 023810 (2010).

42. G. Gui, N. J. Brooks, H. C. Kapteyn, M. M. Murnane, C. T. Liao, Second-harmonic generation and the conservation of spatiotemporal orbital angular momentum of light. *Nat. Photonics* **15**, 608–613 (2021).

43. X. Liu, C. Liang, Q. Cao, Y. Cai, Q. Zhan, Ultrafast bursts of tailored spatiotemporal vortex pulses. arXiv. arXiv:2407.19747 [physics.optics] (2024).
